# Ethyl acetate extract of *Ruta graveolens*: a specific and potent inhibitor against the drug-resistant EGFR_T790M mutant in NSCLC

**DOI:** 10.3389/fphar.2025.1570108

**Published:** 2025-04-29

**Authors:** Vikas Kumar, Leena Chauhan, Deepa Singh, Akash Kumar, Rajkumar Kulandaisamy, Tushar Kushwaha, Kamal Baswal, Rajan Singh, Saroj Kumar, Shivajirao L. Gholap, P. Hariprasad, S. Babu Dadinaboyina, Jagadeshwar R. Thota, Deepak Sehgal, Mohan B. Appaiahgari, Krishna K. Inampudi

**Affiliations:** ^1^ Department of Biophysics, All India Institute of Medical Sciences, New Delhi, India; ^2^ Centre for Rural Development and Technology, Indian Institute of Technology Delhi, New Delhi, India; ^3^ Department of Life Sciences, School of Natural Sciences, Shiv Nadar Institution of Eminence, Gautam Buddha Nagar, Uttar Pradesh, India; ^4^ Chemistry Department, Indian Institute of Technology Delhi, New Delhi, India; ^5^ Department of Analytical and Structural Chemistry, CSIR-Indian Institute of Chemical Technology, Hyderabad, India; ^6^ Laboratory for Multidisciplinary Research in Virology, Yenepoya (deemed to be University), Mangalore, India

**Keywords:** Lung cancer, NSCLC, EGFR_T790M mutation, *Ruta graveolens*, ethyl acetate extract

## Abstract

Lung cancer, the second leading cause of cancer mortality, requires the development of novel therapeutic strategies due to emerging drug resistance and toxicity. With this objective, the present work explored the therapeutic potential of *R. graveolens* leaf extracts against EGFR_T790M-mediated drug resistance in NSCLC. To this end, we evaluated the functional and therapeutic potential of a panel of polar and non-polar solvent extracts using various *in vitro* assay systems. Among the extracts tested, EAE exhibited superior kinase inhibitory activity, which was more pronounced against the EGFR_T790M mutant phenotype. Accordingly, EAE exhibited a favorable cytotoxicity profile and potent growth inhibition of EGFR_T790M-positive NSCLC cells, as evident from its superior IC_50_ values in this cell type. Flow cytometry analysis further validated its inhibitory effects on the cell cycle and, well-supported by the data from the TUNEL assay, suggested induction of apoptosis in EAE-treated cells in a dose-dependent manner. Finally, mechanistic studies in EAE-treated cells showed that these outcomes were due to concentration-dependent inhibition of EGFR phosphorylation at Tyr1068 and Tyr1173. Importantly, this inhibition was consistently more pronounced in H1975 cells expressing the EGFR_T790M mutant phenotype. Further, pull-down assays, followed by mass spectrometry analysis, identified the most promising molecules within EAE. Together, the study highlighted the therapeutic potential of EAE from the leaves of *Ruta graveolens* for treating EGFR_T790M-mediated drug resistance in lung cancer.

## 1 Introduction

Cancer is the second leading cause of death globally after cardiovascular disease. It is responsible for high mortality, with an estimated 9.7 million deaths in 2022 (Bray et al., 2024). Recent research suggests that lung cancer is the most frequently diagnosed cancer worldwide. It accounts for nearly 2.5 million new cases annually and represents 12.4% of all cancers. With approximately 1.8 million deaths, it is the leading cause of cancer-related fatalities, accounting for 18.7% of all cancer deaths (Bray et al., 2024). Histopathologically, lung cancer is classified into SCLC and NSCLC (Spira, 2004). SCLC is defined by its inclination for early metastases and a rapid doubling time, with a 5-year survival rate of only 3% of patients (Simon and Wagner, 2003). Any epithelial lung cancer other than the SCLC is called NSCLC. It accounts for ∼85–90% of all lung cancers, with an overall 5-year survival rate in 15%–17% of patients (Siegel et al., 2023). Mutations in the intracellular tyrosine kinase (TK) domain of EGFR have been reported in the pathogenesis of several human malignancies, including NSCLC (Hirsch and Bunn, 2009; Inamura et al., 2010). Further, EGFR acts as a biomarker and a coherent target for treating NSCLC (Politi and Lynch, 2012).

**FIGURE 1:**
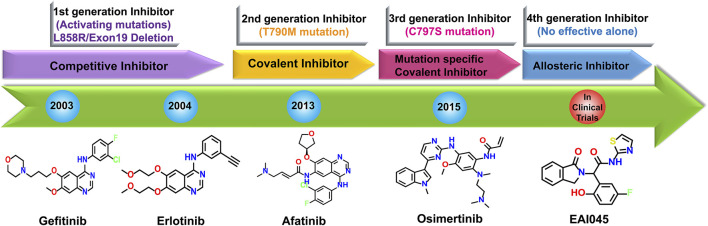
Chronological development of EGFR tyrosine kinase inhibitors.

Mutations in the EGFR kinase domain increase its catalytic activity and act as oncogenic drivers. Function-enhancing mutations in the EGFR gene are more prevalent in exons 18–21, which span the TK domain (Hodoglugil et al., 2013). The most effective approach in NSCLC therapy is targeting the EGFR signaling using TK inhibitors (TKI) (Bartholomew et al., 2017; Shah and Lester, 2020; Zhang et al., 2024; Zhou et al., 2009). Several TKIs were designed against mutant EGFR, among which erlotinib, gefitinib, afatinib, and osimertinib received FDA approval (Nan et al., 2017) (Figure 1). Patients who are initially responsive to EGFR TKIs eventually acquire resistance after 10–16 months of clinical benefit (Herst and Berridge, 2013). Moreover, 20%–30% of NSCLC patients show poor clinical response to EGFR TKIs (Wang et al., 2016). Median progression-free survival after the initiation of TKI treatment among NSCLC patients is about 10 months (Poh et al., 2024). This has been attributed to acquired drug resistance caused by mutations in the EGFR_TK, primarily the EGFR_T790M mutation (Ting et al., 2019). The EGFR_T790M mutation confers resistance to first-generation TKIs by increasing the affinity of TK for ATP, thereby competing out TKIs. Osimertinib, an FDA-approved third generation TKI, has potent inhibitory activity against EGFR_T790M-positive NSCLC but is associated with several adverse events, including an increased risk of cardiovascular diseases (Nishino and Hatabu, 2017). Further, high treatment costs and development of resistance against Osimertinib due to C797S mutation in EGFR (Guo et al., 2023; Thress et al., 2015; Song et al., 2016) limit its clinical use. Accordingly, there is an urgent need to develop novel therapeutics against NSCLC that comprehend the intratumoral EGFR heterogeneity, overcome TKI resistance, and cause milder or no adverse effects in the treated population. Natural products, such as plant secondary metabolites, possess tyrosine kinase inhibitory properties (Baier and Szyszka, 2020) and are promising candidates against drug-resistant EGFR kinases. Among the various natural sources explored in the literature, *Ruta graveolens* has demonstrated broad medical applications in treating multiple disorders, including lung cancer (Varamini, Soltani, and Ghaderi, 2009). A previous study by Fadlalla et al. reported that *R. graveolens* extract suppressed cancer cell proliferation by activating DNA damage pathways and inhibiting *Akt* activation (Fadlalla et al., 2011). Another study of *R. graveolens* extract showed the induction of cell death in glioblastoma cells and neural progenitors, but not neurons, through AKT and ERK 1/2 Activation (Gentile et al., 2015). Given that AKT and ERK are intermediate effectors of EGFR, we hypothesized that *R. graveolens* extracts may possess anti-EGFR kinase activities. We also sought to determine the specificity of these extracts for the drug-resistant EGFR_T790M mutant.

*Ruta graveolens* L. [Rutaceae] commonly known as rue or herb-of-grace, belongs to the Rutaceae family and is native to the Balkan Peninsula (Spence and Spence, 2023). Sterols of *R. graveolens* showed anticancer activity in the human lung cancer (A549) cell line with 81% inhibition at 100 μg/mL (Baker et al., 2017) and in the human epidermoid carcinoma (A431) cell line (Aherne and O’Brien, 2000; Réthy et al., 2006). *Ruta graveolens* is known for its analgesic and anti-inflammatory properties and has shown potential for selectively sensitizing cancer cells to apoptosis while sparing normal cells (Jinous, 2012). Among the metabolites reported so far in the literature, *R. graveolens* contains a diverse range of bioactive metabolites, including furanocoumarins, carotenoids, anthraquinone, furanoquinolones, and alkaloids (Kuzovkina, Al’terman, and Schneider, 2004; Hale et al., 2004; Eickhorst, DeLeo, and Csaposs, 2007). Rutin, which was first isolated from its leaves, and quercetin are the active flavonoids of *R. graveolens* (Pathak et al., 2003). Among these, psoralens, a subgroup of furanocoumarins, are well known for their ability to induce DNA interstrand cross-links upon exposure to ultraviolet (UV) light (DE RIE et al., 1995; Vogelsang et al., 1996).

**FIGURE 2:**
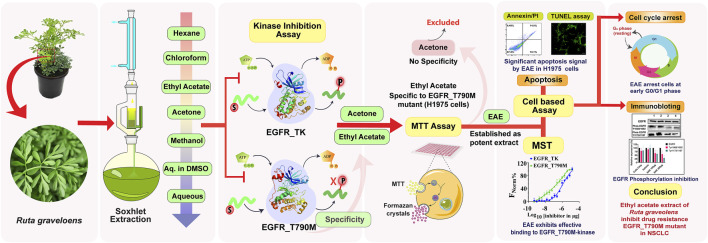
Graphical abstract illustrating the methodology and the bioactivity of EAE of *Ruta graveolens* against drug-resistant EGFR-mediated NSCLC.

Based on the available literature, this study aimed to identify bioactive molecules from the leaves of *R. graveolens* with targeted efficacy against drug-resistant EGFR_T790M mutant lung cancer. Accordingly, the study (Figure 2) investigated the effectiveness of various solvent extracts of leaves from *R. graveolens* against the EGFR_T790M drug-resistant phenotype of NSCLC. Among all the solvent extracts, EAE exhibited higher potency for the EGFR_T790M mutant phenotype. Mechanistically, EAE inhibited EGFR phosphorylation at Tyr1068 and Tyr1173, suggesting its action within the EGFR kinase domain. Pulldown assays followed by mass spectrometry analysis identified specific inhibitors unique to EGFR_T790M.

## 2 Materials and methods

### 2.1 Cloning, expression, and purification of recombinant EGFR_WT and EGFR_T790M

Human EGFR_TK coding for the target region (residues 696-1022) was amplified from the human EGFR Addgene vector (Cat #11011) and cloned into pFastBac HT-B plasmid with N-terminal 6X-His-tag (pF-EGFR_WT_696-1022_) (Greulich et al., 2005). The EGFR_T790M mutant (pF-EGFR_T790M) was generated from this clone using the mutant primers by site-directed mutagenesis. Recombinant clones were transpositioned into DH10Bac cells harboring bacmid DNA to generate recombinant Bacmid DNA. Purified recombinant EGFR_TK and EGFR_T790M bacmids were transfected into Sf21 cells, and the recombinant viruses were rescued from the harvested culture supernatants. Plaque-purified recombinant virus clones were used to infect fresh monolayers of Sf21 cells to produce virus stocks for large-scale virus cultures. Infected cell pellets produced in high-density ExpiSf9™ cells were lysed in the reaction/storage buffer containing 25 mM Tris, 150 mM NaCl, 5 mM BME pH 8.0, and 10% Glycerol. The recombinant EGFR_TK and EGFR_T790M proteins were purified using a 2-stage purification method involving Ni-NTA affinity column chromatography followed by size-exclusion chromatography with 16/600 75 PG column (GE). The purity of the recombinant proteins was confirmed by PAGE and Western blot. Purified proteins were stored at −80°C in the storage buffer described above.

### 2.2 Cell cultures

Given the commercial unavailability of a cell line harboring only the EGFR_T790M mutation, the H1975 cell line (ATCC Cat #CRL-5908), that harbors T790M/L858R mutations, was used as a suitable alternative to determine the efficacy of plant extracts against EGFR_T790M(Chen et al., 2023; El Kadi et al., 2018; Liu et al., 2021; Liu et al., 2020; Walter et al., 2013; Zhao et al., 2015; Zou et al., 2013). A431 cell line overexpressing the EGFR_WT (Merlino et al., 1984), A549 (kRAS Lung Cancer) (Giard et al., 1973) and Jurkat (T4 Lymphoma, EGFR Negative) (Abraham and Arthur, 2004) cell lines were kind gifts from Prof. Deepak Sehgal’s laboratory (Shiv Nadar University, Noida, India), whereas H1299 (nRAS-driven lung cancer) (Ohashi et al., 2013) cell line was a kind gift from Dr. Prabhat Malik (AIIMS, New Delhi, India). All the cell lines were maintained as per the suppliers’ recommendations.

### 2.3 Preparation of *Ruta graveolens* leaf extracts

The *R. graveolens* plant used in this work was cultivated and preserved at the Applied Secondary Metabolite Laboratory nursery at the Centre for Rural Development and Technology, IIT Delhi, India. Initially, Online flora (https://indiaflora-ces.iisc.ac.in/FloraKarnataka/herbsheet.php?id=3979&cat=1) was used to identify the plant. Further, the identity was confirmed by Dr. Sampat Kumar, Lecturer in Biology (Taxonomist), Davangere Government Pre-university College, Karnataka, India, and Dr. Hariprasad P., Associate Professor, CRDT, IITD, Delhi, India. The specimen is maintained in the Applied Secondary Metabolite lab, IIT Delhi, with voucher no. CRDT/ASM/179-WP. The above-ground portion of the mature plants was removed in June and July 2022 before the blossoming using a sterilized stainless-steel knife. The leaves of the gathered plant specimens were excised from stems using a sterile surgical blade. The leaf samples were desiccated in a hot air oven at 40°C ± 2°C for 48 h and coarsely pulverized using a mechanical blender (Prestige Stylo mixer grinder, 750 w, India). Fifty grams of the powder were placed in a thimble and subjected to solvent extraction using a Soxhlet apparatus with a processing capacity of 500 mL. The metabolites in the pulverized powder were extracted successively using six solvents in order of increasing polarity: hexane, chloroform, ethyl acetate, acetone, methanol, and water. Briefly, Soxhlet was operated at 45 °C for hexane, chloroform, and ethyl acetate and run for 6 h for each solvent. In the case of acetone, methanol, and water, the heating was maintained at 50°C and ran for 10 h. At reduced pressure, the solvent extracts were concentrated with a rotary evaporator (Hahn vapor, HS-2005S, 200–240 V, Korea). The water bath temperature and vacuum pressure for hexane (45°C, 60 mmHg), chloroform (45°C, 60 mmHg), ethyl acetate (45°C, 60 mmHg), acetone (45°C, 80 mmHg), methanol (50°C, 80 mmHg) and water (50°C, 40 mmHg) was set. The condenser temperature was set to 4°C for all solvents used. Subsequently, the remaining solvents were eliminated by spreading the samples as a thin layer on a glass plate and placing them in a vacuum oven (Thermo Scientific, Model–Lab Line, 3618-1CE) set at −90 kPa, 40°C ± 2°C for 24 h. The desiccated extracts were reconstituted in dimethyl sulfoxide (DMSO) at 500 mg/mL concentration for further use.

### 2.4 Kinase assay

The kinase activity of purified EGFR_TK and EGFR_T790M mutant protein was assayed using the ADP-Glo kinase assay kit (#V9102 Promega, Madison, WI) (A. Wang et al., 2017) according to a previously optimized protocol (Aiebchun et al., 2021; Bajaj et al., 1987; Bernas and Dobrucki, 2002; Brignola et al., 2002) In a 32 μL reaction volume. Peptide C (RAHEEIYHFFFAKKK), a substrate for EGFR, (Engel et al., 2013) was purchased from Pepmic Co. Ltd., China. Concentrations of the EGFR variants, the peptide substrate, and the ATP were optimized (Supplementary Figure S1) for a reaction time of 60 min at 37°C. Optimized conditions were used to set up the kinase reactions, followed by adding ADP-Glo reagent for 40 min to deplete all the unused ATP, leaving only the ADP produced from the phosphorylation reaction. Next, 16 μL per reaction of the kinase detection reagent was added to convert ADP to ATP, and, subsequently, luminescence was measured at 560 nm (SpectraMax i3x Multi-Mode Microplate Reader). The data was plotted to calculate respective IC_50_ values for different extracts using the GraphPad Prism v9.3.1.

### 2.5 Cell viability assay

Semi-quantitative colorimetric MTT assays were performed to determine cell viability and cytotoxicity of the plant extracts. The assay involved seeding ∼10^4^ cells per well of a 96-well culture plate and incubating the same at 37°C in a CO_2_ incubator to produce ∼80% confluent cell monolayers. Serial dilutions of plant extracts and DMSO vehicle control were prepared using the same dilution factor. Untreated control cells were treated with media only. All cells, including controls, were then incubated for 48 h. After 48 h of incubation, 100 μL of MTT reagent at 0.45 mg/mL was added per well and incubated for 4 h. Next, 100 μL DMSO was added to dissolve the formazan crystals over 2 h of incubation. The plates were read at 570 nm, background absorbance was subtracted, and the data was analyzed to determine the percent cell viability in treated versus untreated cells. Cell viability data was plotted against extract concentrations, and the IC_50_ values were derived from the dose-response curves.

### 2.6 Microscale thermophoresis binding assay

A Microscale thermophoresis assay was set up to determine the binding affinity of the selected extracts for EGFR_TK and EGFR_T790M kinases. Briefly, 200 nM of purified EGFR and its variant were fluorescently labeled with red Tris-NTA his-tag labeling dye (Nano temper Technologies, United States) as recommended. The mixture was kept in the dark for 30 min, followed by centrifugation at 15,000 × *g* for 10 min at 4°C. Two-fold serial dilutions of the extract were mixed, each with 200 nM of fluorescently labeled protein at a 1:1 ratio. MST was run on the Monolith NT.115 instrument at an ambient temperature of 25°C, with instrument parameters set to 50% LED and medium MST power. The samples were loaded into capillaries and placed inside the capillary tray of Monolith NT.115 MST instrument (nano temper technologies). The data generated for each extract, a protein combination from triplicate samples, was analyzed using Monolith NT.115 analysis software (Nanotemper Technologies).

### 2.7 Flow cytometry assays

Cell viability and induction of apoptosis were analyzed by flow cytometry using Alexa fluor 488 Annexin-V/Dead cell apoptosis kit (Cat # 13241, Invitrogen). For this, ∼3 × 10^5^ cells were plated in each well of a 6-well plate to form sub-confluent monolayers and then treated with the selected plant extracts at pre-determined concentrations. Cells only, as untreated controls, were treated with media, whereas the vehicle controls had media supplemented with DMSO in the same proportion as the extracts and incubated for 48 h. After removing the culture supernatant, cells were treated with trypsin-EDTA, and the cell pellets were obtained by centrifugation. Cells were resuspended in Annexin binding buffer containing Annexin-V-Alexafluor 488, as described in the kit. Similarly, cell cycle inhibition by the selected plant extracts was analyzed using the Propidium Iodide (PI) reagent (Himedia SKU: ML067) according to the manufacturer’s protocol. The data was analyzed and plotted using CytExpert 2.5 software (Accuri Cytometers) and FloJo v10.0.

### 2.8 TUNEL [terminal deoxynucleotidyl transferase (TdT) dUTP Nick-end labeling] assay

The assay was performed using “Dead Cell Apoptosis Kit with Annexin V Alexa Fluor™ 488 and Propidium Iodide (PI) (Cat# V13241)”. Measurement of DNA fragmentation in apoptotic cells relies on TdT-mediated attachment of fluorescently labeled deoxynucleotides to the 3′-OH terminus of DNA breaks. For this, cells were seeded in a 6-well plate at ∼3 × 10^5^ cells per well, grown to sub-confluency, and then treated with a predetermined concentration of the selected plant extract(s). Plates were incubated in a CO_2_ incubator for 48 h, monolayers rinsed with 1× Dulbecco’s PBS (DPBS), fixed in 4% paraformaldehyde for 25 min at 4°C, again rinsed twice with PBS and permeabilized with PBS-T containing 0.2% Triton X-100. Subsequently, cells were equilibrated at 37°C in a humid chamber for 60 min with TdT, and the reactions were terminated by adding 2× SSC, followed by three PBS rinses to remove excess fluorescein-12-dUTP. Necrotic cells were PI-labelled (#ML067 Himedia), while the live cells were labeled with DAPI for 15 min, followed by a rinse with nuclease-free water. Slides were mounted using one drop of anti-fade solution (Molecular Probes, Cat. #S7461) and analyzed under a fluorescence microscope for AlexFluor™ 488 at 520 nm, for PI at >620 nm and DAPI at 460 nm. All the images were captured on a Nikon Ti2E A1R MP Laser Scanning Confocal Microscope at a magnification of ×20 (Plan Apochromat 20X/0.75 NA).

### 2.9 Immunoblotting

Cells treated with/without EAE and lysed with RIPA buffer (#R0278, Sigma), were subjected to sonication for 2 min, and then centrifuged at 15,000 × *g* for 15 min at 4°C. The supernatant was collected and quantified using Bradford reagent (# 5000006 Bio-rad). Equal concentrations of proteins were resolved on 12% SDS-PAGE, transferred onto PVDF membranes (#IPVH00010 Merck), and treated with 5% non-fat milk powder in PBS-T. Primary antibodies were used at 1:1,000, while the peroxidase-conjugated anti-rabbit and anti-mouse IgG antibodies were used at 1:3,000 dilution (Bio-Rad). The blots were developed using the Chemiluminescent HRP Substrate (ECL kit, Bio-Rad). Antibodies used in the immunoblot assay were precision mouse anti-EGFR (#VMA00982 Bio-Rad), phosphor-EGFR (Tyr1068) [1092] (#AF3045, Affinity Biosciences), phosphor-EGFR (Tyr1173) [1197] (#AF3048, Affinity Biosciences), Goat-α-rabbit (#64524573, Bio-Rad), α-mouse (#32430, Bio-Rad), and Rabbit-α-GAPDH (#VPA00453, Bio-Rad) antibodies. Finally, using ImageJ, blots were subjected to densitometry analysis to quantify EGFR phosphorylation.

### 2.10 Pull-down assay and mass spectrometry analysis

#### 2.10.1 Pull-down assay

To identify metabolites in EAE that specifically bound to EGFR_T790M and EGFR_TK, a pulldown experiment was performed using a 0.2 ml column containing 50 µl Ni-NTA resin. The column was washed with deionized water and three column volumes of binding buffer (50 mM Tris-HCl, pH 8.0; 150 mM NaCl; and 2 mM DTT). Then, 200 µg of purified EGFR-TK and, in a separate experiment, 200 µg of purified EGFR_T790M were loaded onto the column and incubated with rotation for 2 hours at 4°C. Unbound protein was removed with a wash buffer. Subsequently, 200 µg of plant extract (EAE) was added to the column and incubated overnight with constant rotation at 4°C. The following day, the column was washed five times with wash buffer, and the molecules bound to the protein were eluted with 100 µL of 50 mM citrate buffer, pH 3.0. The eluted plant extract fractions were lyophilized and used for mass spectrometry analysis.

#### 2.10.2 LC-MS/MS analysis

The lyophilized plant extracts from the pull-down assay were reconstituted in 400 µL of methanol and filtered through a 0.22 µm syringe filter before being injected into the LC-MS. The LC-ESI-HRMS and MS/MS analysis of plant extracts were carried out on Orbitrap Exploris 120 mass spectrometer (Thermo Fisher Scientific, United States) coupled to Vanquish UHPLC (Thermo Fisher Scientific, United States). An autosampler introduced an injection volume of 10 µL of the sample into the mass spectrometer. The chromatographic separation was achieved using the Aquity UHPLC BEH C18 column (50 × 2.1 mm × 1.7-µm, M/s. Waters India Pvt. Ltd.), column temperature: 40°C and auto-sampler temperature: 7°C. A two-component mobile phase was used at a flow rate of 0.4 mL/min, where 0.05% formic acid in ultrapure water was used as mobile phase A and 0.05% formic acid in acetonitrile was used as mobile phase B, respectively. The optimized gradient program was set as follows: (time in min/% of Mobile phase B) 0–2/2, 2–10/98, 10–17/98, 17–20/2, and 20–22/2.

Mass spectral data was acquired in both the positive and negative ionization modes using the Heated-electrospray ionization (H-ESI) as an ionization source. The typical MS conditions were: the capillary voltage was set at 3500 V and 2500 V for positive and negative ionization modes, respectively; nitrogen (N_2_) gas was used as the sheath gas and auxiliary gas, and their flow was maintained at 50 (Arbitrary value) and 10 (Arbitrary value), respectively. The ion transfer tube and vaporizer temperatures were set at 300°C and 350°C, respectively; the CID gas pressure was set at 1.5 mTorr. The mass spectrometer was operated in full scan mode with data-dependent MS2 (ddMS2) acquisition. The full scan parameters are: scan range (m/z) 75–1,000, orbitrap resolution 60,000, RF lens (%) 70, and profile data acquisition. The ddMS2 parameters were as follows: isolation window (m/z) 2, collision energy type- normalized, HCD collision energies (%) were kept at 20, 30, and 40 eV, orbitrap resolution was set to 15,000 (FWHM), centroid-acquisition data type, peak filters: intensity threshold 2. e5, singly charged charge state, dynamic exclusion duration = 5 s. The acquired raw data was further processed using Compound Discoverer (v3.3, Thermo Fisher Scientific) for untargeted metabolite identification. Identification was based on accurate mass, retention time, and MS/MS spectral matching against online databases (ChemSpider, mzCloud, mzVault, MassList, HMDB). The resulting table (m/z, retention time, ion adduct, peak intensity) was refined by removing redundant ions, duplicates, and ions with CV > 15%. Finally, chromatograms (Supplementary Figures S2A,B, S3A,B, S4A,B) and MS/MS spectra quality were manually inspected to ensure accurate metabolite identification.

### 2.11 Statistical analyses

For statistical analyses, experimental data for each point was obtained from at least three replicates and represented as data mean ± standard deviation. The IC_50_ concentrations of the extracts were derived by modeling the data sets using a non-linear regression model. Statistical significance between different experimental groups was calculated by performing two-way ANOVA, paired t-tests, and one sample t-test. A *p-value* of <0.05 is considered significant in all the experiments. All the statistical analyses were performed using the *built-in* statistical methods available in GraphPad Prism version 9.3.1. (GraphPad, United States).

## 3 Results

Lung Cancer remains one of the significant challenges to combat, especially with the development of drug resistance, including EGFR_T790M mutation. This study is focused on finding plant-based therapeutic solutions to overcome the EGFR_T790M mediated drug resistance in NSCLC. In this direction, extracts from *R. graveolens* have been shown to target EGFR signaling pathways through ERK/AKT inhibition. Accordingly, in the present study, a variety of solvent extracts from R. graveolens were prepared by standard Soxhlet extraction methods. Subsequently, recombinant EGFR variants were produced using the Baculovirus expression system, followed by the development of kinase-inhibition assays. From these assays, potential extracts having specificity for the EGFR_T790M mutant were identified and characterized in cell-based assays for various anticancer properties to establish the mode of action as well as to identify potential bioactive molecules mediating inhibition of EGFR phosphorylation.

### 3.1 EAE and acetone extract shows selectivity for EGFR_T790M mutant kinase

**FIGURE 3:**
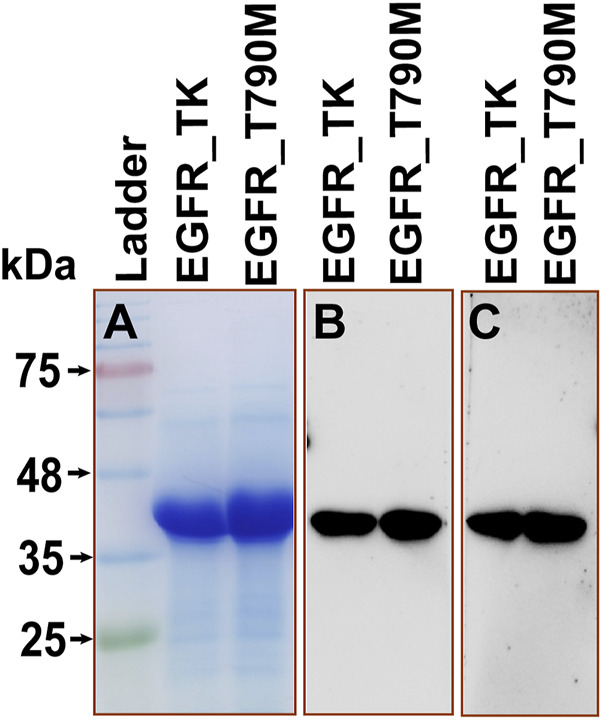
Purified EGFR_TK and EGFR_T790M proteins **(A)** Coomassie-stained 12% SDS-PAGE **(B)** Immunoblot with anti-His (1:3,000 dilution) antibody, **(C)** Immunoblot with anti-EGFR-TK-specific mAb (1:1,000 dilution).

**FIGURE 4:**
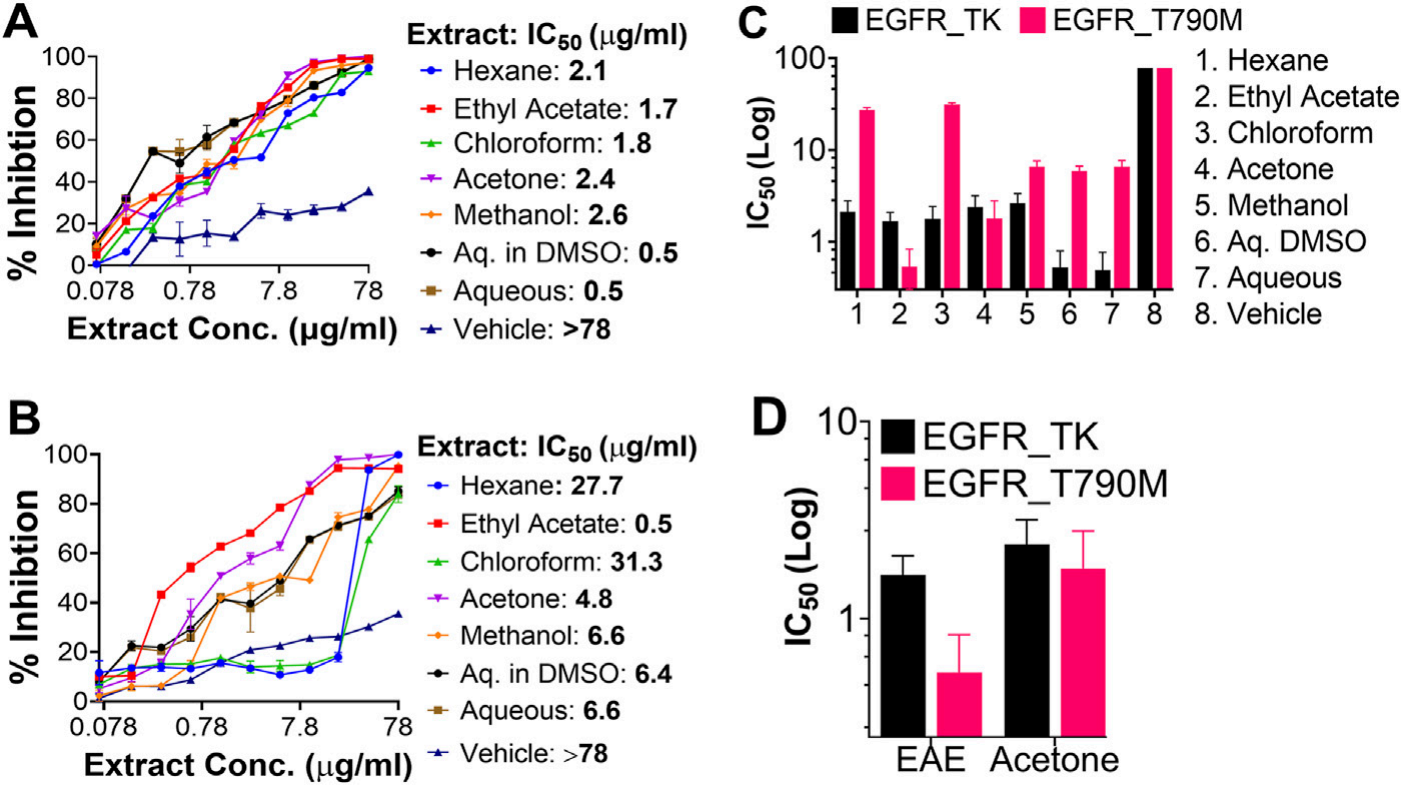
The kinase-inhibitory potential of different solvent extracts against EGFR variants. Dose-response curves were generated from the titration data, and their respective IC_50_ values were derived using GraphPad Prism v.9.3.1. In each graph, the *X*-axis represents the concentration of the extract in a linear scale. In contrast, the *Y*-axis represents the percentage of cell viability (0–100). **(A)** Inhibition of EGFR_TK activity, **(B)** Inhibition of EGFR_T790M activity, **(C)** Bar-graph representation of the IC_50_-values of each solvent extract against the two EGFR variants. **(D)** Bar-graph representation of the IC_50_-values of EAE and Acetone extract. Here, we show the superior selectivity of EAE exhibiting a 3.4-fold difference between the kinase activities of EGFR_TK and EGFR_T790M. The data shown here represents three independent experiments, and the error bars represent the standard deviation. A *p*-value of <0.05 is considered significant for all statistical analyses. (n = 3).

EGFR_TK and EGFR_T790M variant Bacmid clones were used to transfect Sf21 cells, recombinant viruses rescued and plaque-purified. Purified clones of each variant were used to produce recombinant proteins from high density ExpiSf9™ cultures. The 2-stage purification process yielded ∼8 mg/L of kinases (Figure 3). The two recombinant EGFR variants were used to optimize the kinase inhibition assays. Optimal reaction conditions for both variants were determined to be 4 μM enzyme, 200 μM substrate, and 500 μM ATP, incubated at 37°C for 1 hour (Supplementary Figure S1). Next, the kinase inhibitory activity of different solvent extracts against the recombinant EGFR variants was investigated under optimized reaction conditions. All the solvent extracts exhibited notable but variable inhibitory activities against the two EGFR variants compared to the vehicle control. Of note, the EAE exhibited potent inhibitory activity against both the EGFR variants. Importantly, its activity against the EGFR_T790M was at least 3.4-fold higher than that against the EGFR_TK. Acetone extract also exhibited similar, but lesser, inhibitory activity against the two EGFR variants compared to the EAE. While the results from these assays suggested superior selectivity of EAE against the EGFR_T790M, both EAE and acetone extract were considered for further cell-based biological characterization. All other extracts that exhibited higher selectivity against EGFR_TK were excluded from further investigations. Relative inhibition of enzyme activities by different plant extracts against wtEGFR_TK and EGFR_T790M, and their corresponding IC_50_ values are shown in Figures 4 A-C. The 3.4-fold higher inhibitory effect of EAE against the EGFR_T790M mutant compared to that against the EGFR_TK, which highlights the selectivity of EAE for the mutant EGFR, is presented in Figure 4D.

### 3.2 EAE is specific against drug-resistant lung cancer cell line

**FIGURE 5:**
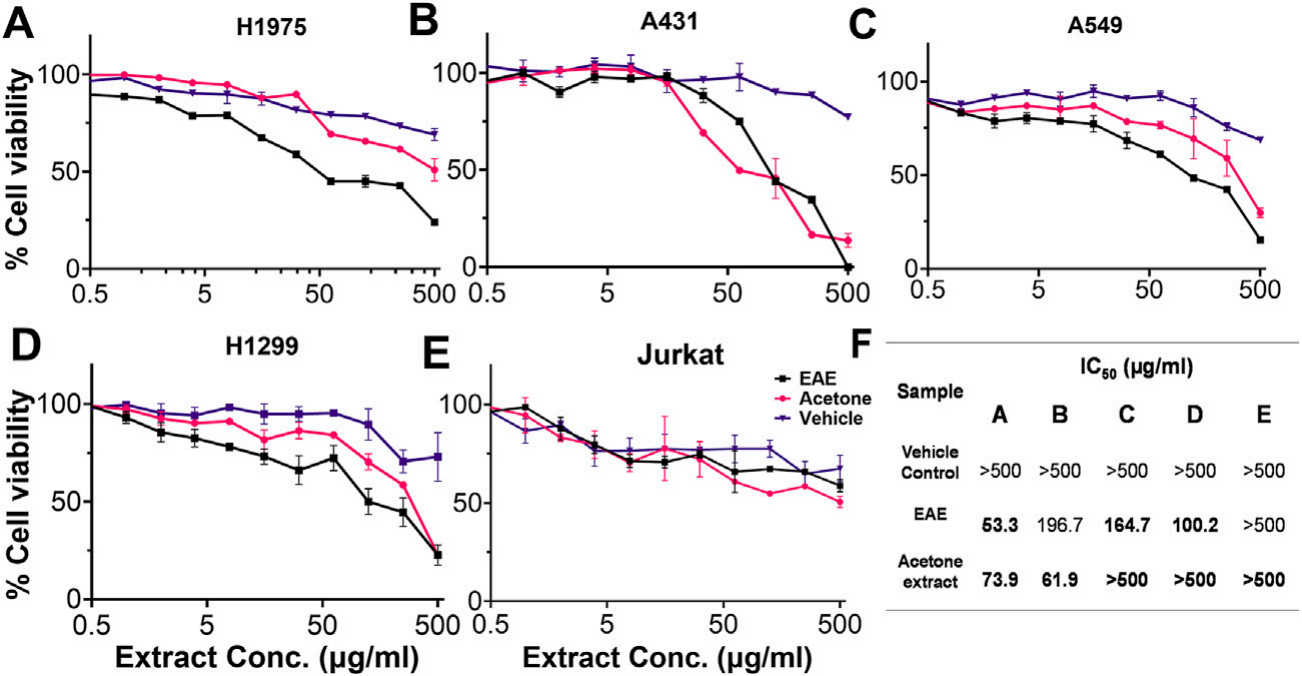
MTT assay showing cytotoxic effects of EAE and Acetone extract on various cell lines. Dose-response curves (*X*-axis: extract concentration; *Y*-axis: % cell viability) and IC_50_ values (calculated using GraphPad Prism v.9.3.1) are shown for **(A)** H1975, **(B)** A431, **(C)** A549, **(D)** H1299, **(E)** Jurkat cells. **(F)** Table summarizes IC_50_ values. Data are mean ± SD from three experiments (p < 0.05). (n = 3).

Cell-based assays were performed on different lung cancer cell types and in non-lung cancer cell lines to investigate the biological activity of the selected extracts. EAE and Acetone extract reduced cell viability in a dose-dependent manner in H1975 (EGFR_T790M+), A431 (wtEGFR), H1299 (nRAS-driven Lung Cancer), and A549 (kRAS-driven Lung Cancer) cell lines compared to the non-cancerous EGFR-negative Jurkat cell line (T-lymphocyte) (Figure 5). Notably, EAE demonstrated selective inhibition of EGFR-positive lung cancer cells (Figures 5A–D), and no significant inhibition, even at higher extract concentrations, of the EGFR-negative Jurkat cells was observed (Figure 5E). Like EAE, acetone extract was also effective against all the EGFR-positive cell types except against the A549 cell line. Importantly, with the *p*-value of < 0.0001, the inhibitory activity of acetone extract on the EGFR_WT-positive A431 cell line (IC_50_ = 61.9 μg/mL) was significantly superior to that of EAE (IC_50_ = 196.7 μg/mL) (Figure 5B). However, it was equally effective against the EGFR-negative non-lung cancer Jurkat cell line, suggesting its non-specific cytotoxicity against all cell types (Figure 5E). These results together suggest superior inhibition of EGFR-positive lung cancer cell types by the EAE and its preferential selectivity for the EGFR_T790M mutant phenotype over the acetone extract. Their respective IC_50_ is given in Figure 5F. EAE exhibited 3-fold more potent inhibitory activity against the EGFR_T790M mutant (IC_50_: 53.3 μg/mL) compared to EGFR_WT (IC_50_: 196.7 μg/mL) (Figure 5F), suggesting the presence of an enriched bioactive content in EAE that is more specific against the EGFR_T790M mutant. While acetone extract [IC_50_: 73.9 μg/mL (EGFR_T790M) vs 61.9 μg/mL (EGFR_WT)] showed inhibitory activities against both EGFR variants, the difference in their effective concentrations was relatively lower compared to EAE. In addition, the acetone extract exhibited nonspecific inhibition in EGFR-negative Jurkat cells. Hence, Acetone extract was excluded, and EAE was selected for further experiments.

### 3.3 EAE exhibits a strong binding affinity for the mutant EGFR

**FIGURE 6:**
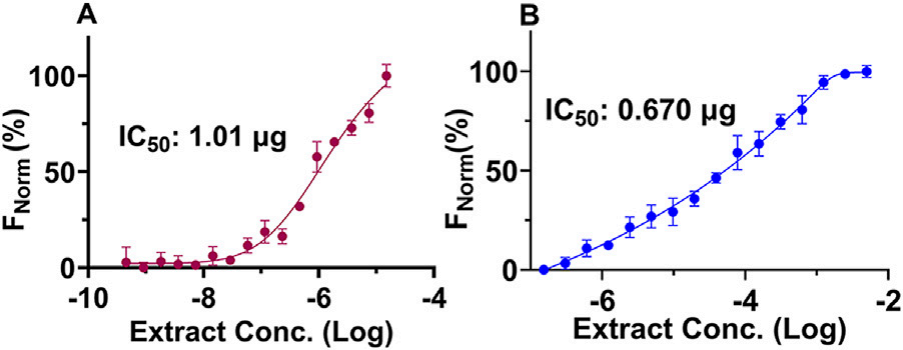
MST analyses of protein-ligand interactions. Two-fold serial dilutions of EAE were incubated with EGFR_TK and EGFR_T790M protein, and the reactions were analyzed on the Monolith NT.115 instrument. The mean of the % normalized fluorescence [F_Norm_ (%), *Y*-axis] for the two EGFR proteins calculated from triplicate experiments was plotted against the EAE concentrations (*X*-axis) to generate the dose-response curves and to derive the *Kd* values. Error bars represent the standard deviation between the three experimental data sets. **(A)** MST binding curve of EGFR_TK with EAE, and **(B)** MST binding curve of EGFR_T790M with EAE. (n = 3).

The MST analysis of ligand-protein interactions provides information on the binding strength between them by analyzing the movement of biomolecules over a temperature gradient. This temperature gradient influences the molecular properties, such as the interacting partners’ charge, size, hydration shell, and conformations. Accordingly, binding affinities of EAE with the purified EGFR_TK and EGFR_T790M were determined. The data from triplicate experiments was used to plot the dose-response curves, and the statistical significance of the differences in binding kinetics was calculated using the unpaired *t-*test with an error margin of 5% (*p* < 0.05). Interestingly, EAE showed stronger interaction with the EGFR_T790M variant with a *K*_*d*_ value of 0.670 µg compared to EGFR_TK, which had a *K*_*d*_ value of 1.01 µg (Figure 6). The results obtained here are well aligned with those from the kinase inhibition assay.

### 3.4 EAE treatment enhances induction of apoptosis

**FIGURE 7:**
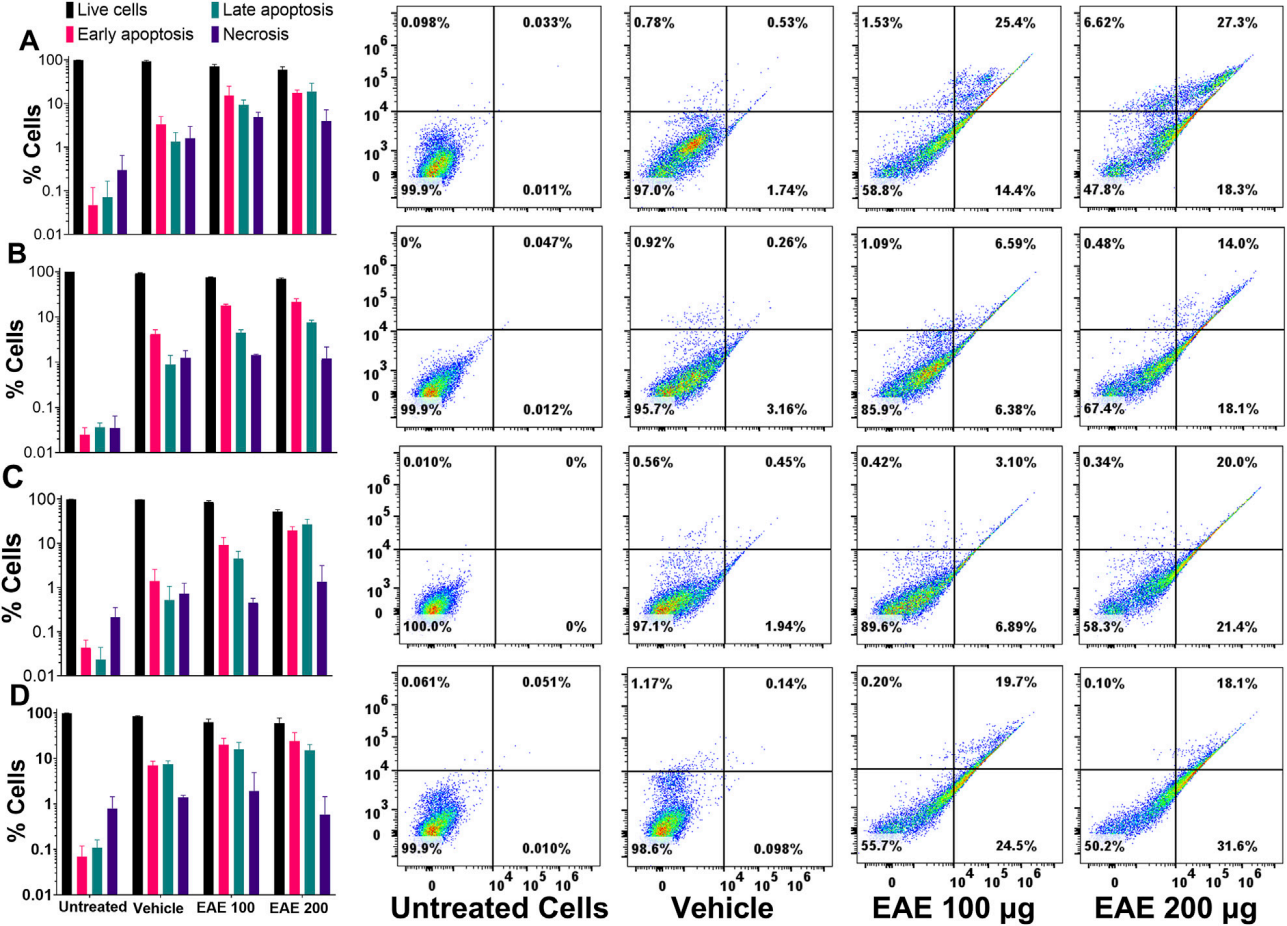
**(A)** H1975, **(B)** A431, **(C)** A549, **(D)** H1299 Cells. The Bar graph on the left and their corresponding four quadrant on the right side, shows the effects of EAE treatment on the induction of apoptotic phenotype and their progression to the necrotic phenotype in different cell types. Different cell types were treated with/without EAE for 48 h, stained with AnnexinV and PI, and analyzed by flow cytometry. Cells positive for the necrotic phenotype were gated in Q1, those in the late apoptotic state were gated in Q2, those in the early apoptotic state in Q3, and healthy cells in Q4. Data was plotted using the GraphPad Prism v9.3.1. Statistical significance was analyzed using the in-built statistical methods. A *p*-value of <0.05 was considered significant, indicating a *p* < 0.0001. The plotted data shown here represent three independent experiments, and the error bars represent the standard deviation between them. (n = 3).

Since IC_50_ values for EAE in different cell types ranged between 50–200 μg/mL, all future studies used 100 μg/mL and 200 μg/mL of EAE. Accordingly, different cell types used in this study were treated with/without 100 μg/mL or 200 μg/mL of EAE for 48 h and subsequently stained with Annexin V/PI. Post-staining flow cytometry analysis revealed a higher rate of apoptosis in EAE-treated cells compared to untreated or vehicle-treated cells (Figure 7). Consistent with the preliminary findings in this study, EAE treatment resulted in a higher percentage of H1975 (EGFR_T790M mutant) cells exhibiting the apoptotic phenotype than A431 cells (EGFR_WT) in a dose-dependent manner. Accordingly, ∼39.8% of H1975 cells were positive for the apoptotic phenotype compared to ∼13% of A431 cells at 100 μg/mL EAE treatment, which increased to ∼45.6% in H1975 cells compared to ∼32.1% of A431 cells at 200 μg/mL (Figures 7A,B). Notably, EAE induces apoptosis in EGFR_T790M cells without progressing to necrosis, while higher doses have less effect on H1975 cells, highlighting its selective apoptotic effect. Contrary to its effects on H1975 and A431 cell types, EAE had a variable impact on the other two lung cancer cell lines, A549 and H1299 (Figures 7C,D). Apoptosis was observed in 10% of A549 cells and 44.6% of H1299 cells treated with 100 μg/mL EAE, compared to 41.4% of A549 cells and 49.7% of H1299 cells treated with 200 μg/mL. Interestingly, irrespective of the EAE dosage, no detectable transition from apoptotic to necrotic phenotype was observed in either A549 or H1299 cells (0.42% at 100 μg/mL vs*.* 0.20% at 100 μg/mL) compared to untreated cells (Figures 7C,D). These results suggest that apoptosis induction was dose-dependent in A549 and H1299 cells. However, only A549 cells exhibited necrotic phenotype, which was also dose-dependent.

**FIGURE 8:**
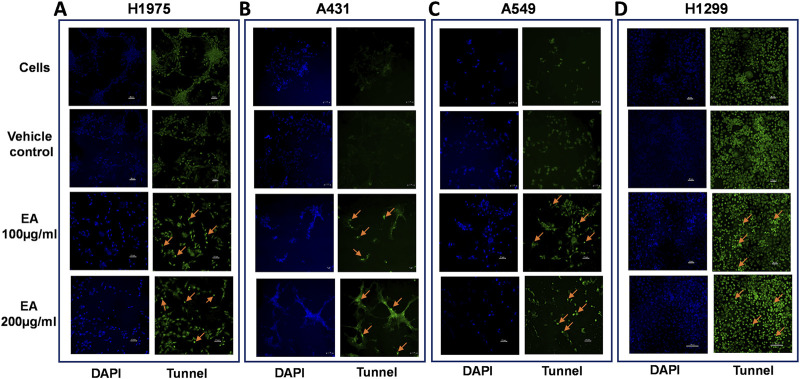
Shows TUNEL staining, indicative of apoptotic state, in different lung cancer cell types. Cells were treated with the selected concentrations (100 and 200 μg) of EAE and stained using the TUNEL assay kit. Different treatment variables are represented on the left. Blue-stained cells in the image represent DAPI staining, and those stained green represent FITC or TUNEL signal. The representative images from one of the three TUNEL assays are shown. TUNEL-positive cells among the fluorescently-stained cells are indicated with an arrow. **(A)** H1975 cells, **(B)** A431 cells, **(C)** A549 cells, **(D)** H1299 cells. (n = 3).

Selective induction of apoptosis in cells expressing EGFR genes after EAE treatment was further confirmed by TUNEL assay. After 48 h EAE treatment with 100 μg/mL and 200 μg/mL, H1975 cells showed a notable increase in staining intensity, indicative of apoptotic activity, compared to untreated and vehicle-treated cells (Figure 8A). Similarly, untreated or vehicle-treated A431 cells hardly showed any staining. However, they showed notable, *albeit* relatively less compared to that in H1975 cells, TUNEL-positivity after EAE treatment in a dose-dependent manner (Figure 8B). Similar to that in the H1975 and A431 cells, A549 cells too showed an increase in TUNEL-positive cells only after the EAE treatment (Figure 8C). On the other hand, control H1299 cells showed intense staining. This staining increased marginally upon EAE treatment (Figure 8D). Overall, these results in different lung cancer cell types agree with the flow cytometry data discussed above on the apoptotic state.

### 3.5 EAE selectively induces G1/G0 phase cell cycle arrest in H1975 cells

**FIGURE 9:**
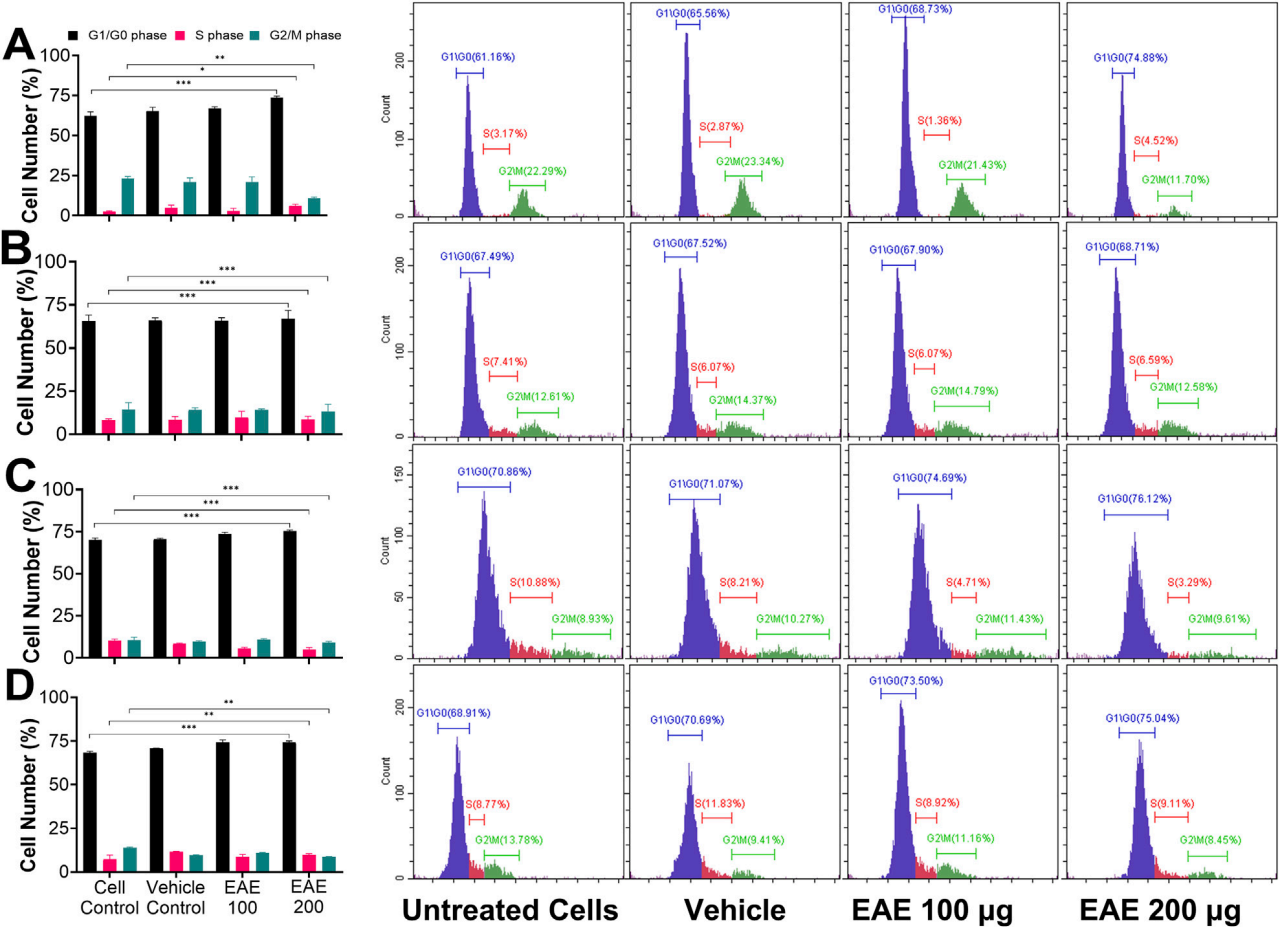
Shows the inhibitory effects of EAE on the cell cycle in different cell types. Cells were left untreated (cell control) or treated with DMSO (vehicle control) or EAE at 100 or 200 μg for 48 h and stained with PI. Cell suspensions were then analyzed on the flow cytometer and quantitated based on the cell cycle phase. These experiments were performed in triplicates, and representative data from one of the replicate experiments is presented here. Cells at the G1/G0, S, and G2/M phases of the cell cycle are colored differently, and their percentages in the total cell population in the sample are indicated. **(A)** H1975 cells, **(B)** A431 cells, **(C)** A549 cells, **(D)** H1299 cells. Figure (Bar graphs) also shows the mean percentages of cells at different cell cycle phases with their respective standard deviations between the triplicate assay data, plotted as a bar graph against the treatment type using Cytoflex software Cytxpert 2.5. A two-sample t-test was performed to estimate the statistical significance, and a *p*-value < 0.05 is considered significant. *, *p* < 0.05; **, *p* < 0.005; ***, *p* < 0.0001. (n = 3).

All four lung cancer cells were treated with DMSO (vehicle) or 100 or 200 μg/mL of EAE for 48 h and analyzed for cell cycle stages by flow cytometry. Data from vehicle-treated cells were compared to those from EAE-treated cells. Irrespective of the cell type, EAE treatment increased the number of cells captured at the G1/G0 phase, which was dose-dependent (Figure 9A). These results suggest that EAE treatment generally causes cell cycle arrest at the G1\G0-phase. However, the effect is relatively more specific to cells expressing the EGFR_T790M mutant phenotype, as none of the other cell types exhibited the trend observed with the H1975 cell line (Figures 9B–D).

### 3.6 EAE inhibits EGFR phosphorylation at Tyr1068/1173 and affects EGFR-mediated signaling

**FIGURE 10:**
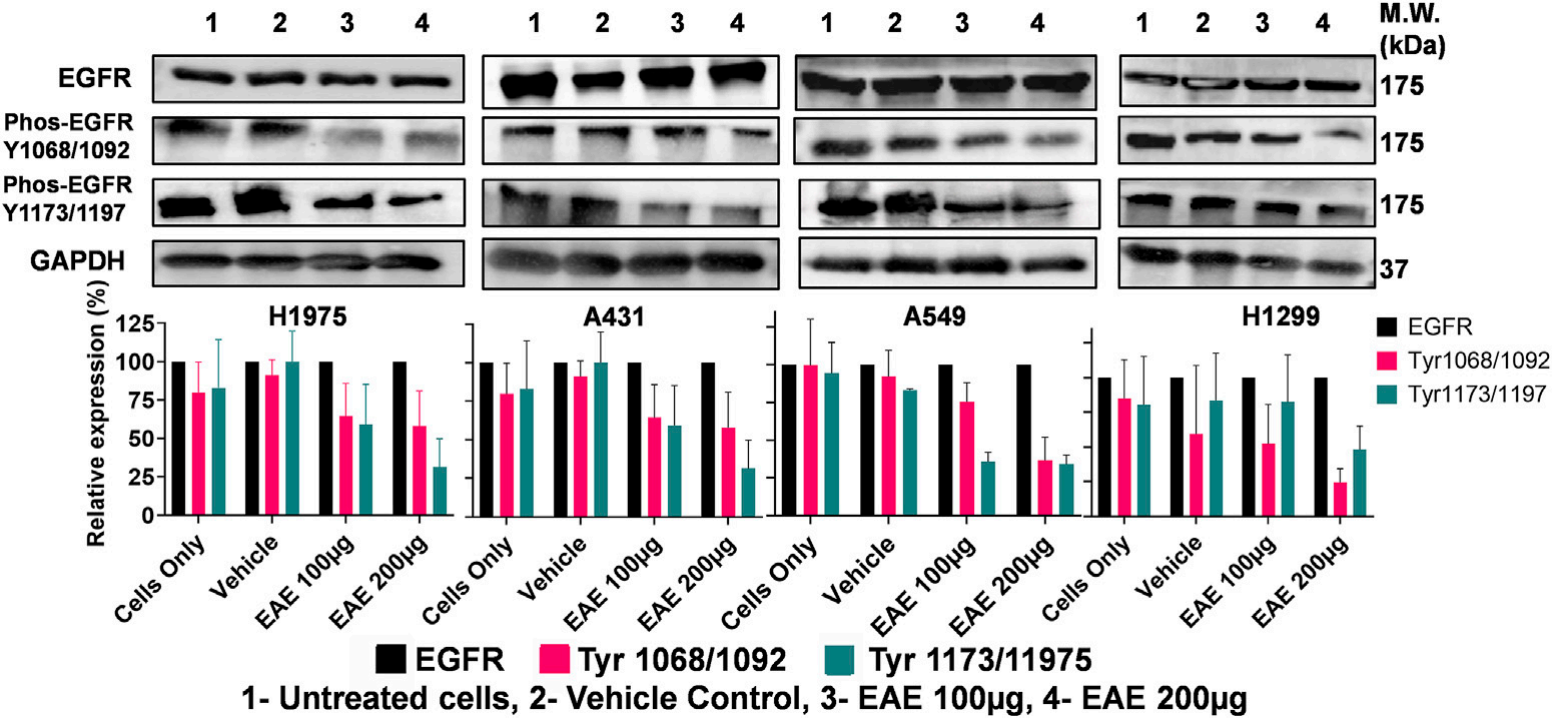
Immunoblot analysis of EGFR phosphorylation inhibition in various cell lines treated with EAE. Panel shows: **(A)** H1975 cells, **(B)** A431 cells, **(C)** A549 cells, **(D)** H1299 cells. Cells were untreated or treated with DMSO (vehicle control) or EAE (100 and 200 μg) for 48 h. Cell lysates were analyzed for EGFR, phospho-EGFR (Tyr1068/1092 and Tyr1173/1197), and GAPDH (loading control) by immunoblotting. The experiment was performed in triplicates, so the densitometry analysis, represented as bar graphs with error bars, showed significant inhibition of EGFR phosphorylation in all cell lines following EAE treatment compared to control expression of EGFR across all cell lines. (n = 3).

Since EGFR phosphorylation is associated with tumor progression, we used Tyr1068/1092 and Tyr1173/1197 as reference sites. These sites were used to study the effect of EAE treatment on phosphorylation and its subsequent effects on the EGFR signaling cascade. Total cell lysates after 48 h EAE treatment were subjected to immunoblotting using mAb specific to unphosphorylated EGFR and phosphorylated EGFR at Tyr1068/1092 and Tyr1173/1197. Phosphorylated and unphosphorylated EGFR levels were normalized to the total EGFR, with basal expression as the reference. All four lung cancer cells consistently expressed the EGFR, but the levels of EGFR phosphorylated at Tyr1068/1092 and Tyr1173/1197 varied between cell types (Figures 10A–D). H1975 cells exhibited a notable reduction in EGFR phosphorylation at both the tyrosine residues in response to both concentrations of EAE (Figure 10A). In contrast, the other three cell types responded differently (Figures 10B–D). However, a general decreasing trend in EGFR phosphorylation is observed across the cell types. It may be noted that inhibition of Tyr1068/1092 phosphorylation leads to the arrest of the EGFR pathway early during the signaling cascade. In contrast, inhibition of Tyr1173/1197 phosphorylation leads to the arrest during the later signaling phases. Accordingly, the more decisive influence of EAE on H1975 cells can be attributed to its dual and more potent effects at both the tyrosine phosphorylation sites in a highly dose-dependent manner. In other cell types, the influence of EAE was weaker, which may be explained by its inhibitory effects predominantly on one of the two tyrosine phosphorylation sites.

### 3.7 Binder molecules specific to EGFR_T790M in EAE

**FIGURE 11:**
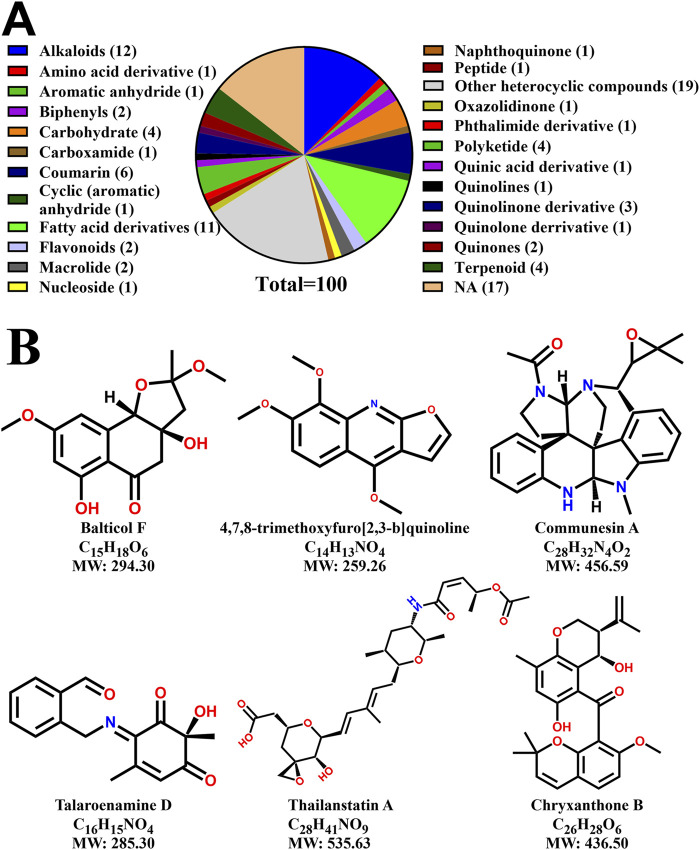
Characterization of EAE and identification of potential EGFR_T790M-specific binders from the mass spectrometry analysis. **(A)** A pie chart showing the most abundant molecules in the EAE, their respective classes and the number of molecules identified in each class, **(B)** Chemical structures of unique binder molecules of EGFR_T790M having drug-like properties, as determined by SWISS-ADME analysis.

To identify the chemical composition of EAE, mass spectrometry analysis was carried out on the whole extract isolated in Ethyl acetate (Supplementary Figures S2A,B). This analysis revealed that EAE comprises several classes of bioactive molecules, such as alkaloids, polyketides, coumarins, terpenoids, flavonoids, quinolines and other classes. The distribution of the top 85 most abundant molecules (Supplementary Mass Spectrometry Data), categorized by molecular class, is illustrated in a pie chart (Figure 11A). To identify specific bioactive molecules within EAE that bind to EGFR_T790M, pull-down assays followed by mass spectrometry analysis were conducted (Supplementary Figures S3A,B), using EGFR_TK (Supplementary Figures S4A,B) as a background control. Following this analysis, molecules common to both the EGFR variants were subtracted to identify binder molecules unique to the EGFR_T790M. From this analysis, we identified 30 molecules having an MS/MS match above 80%, of which 6 molecules were found to have drug-like properties as determined by Swiss-ADME analysis. The chemical structures and the molecular information of these molecules are illustrated in Figure 11B. These include Balticol F, 4,7,8-trimethoxyfuro [2,3-b]quinoline, Communesin A, Talaroenamine D, Thailanstatin A, and Chryxanthone B, which have shown the critical ADME properties and are presented in Supplementary Table S1.

## 4 Discussion

The present study investigated the kinase inhibitory activities of different solvent extracts from the leaves of *R. graveolens* against EGFR_TK and EGFR_T790M mutant. Our results from the kinase-inhibition assays suggested that the EAE was relatively more effective against the mutant, highlighting its selectivity against the drug-resistant NSCLC. Some of the studies in the published literature have explored the therapeutic potential of synthetic molecules targeting the EGFR_T790M(X. Tang et al., 2021; Zhou et al., 2009). Osimertinib, a third generation TKI, have shown such prominent effectiveness (Zhou et al., 2009; Nishino and Hatabu, 2017; Planchard et al., 2015; Song et al., 2016; Goss et al., 2016; Wu et al., 2018). However, the development of resistance and off-target effects made them less effective, necessitating exploring alternative or complementary therapeutic strategies. Unfortunately, while a plethora of information exists on synthetic molecules or purified natural compounds, to the best of our knowledge, studies showing the activity of plant extracts against EGFR_T790M using *in vitro* kinase inhibition assays are lacking. The specificity of EAE for the mutant EGFR was further established through cell-based experiments. Here, while EAE treatment led to a decrease in the viability across different cell types, the effect was relatively more pronounced in H1975 cells that express the EGFR_T790M mutant. Further, there was dose-dependency in the EAE-mediated cytotoxicity in H1975 cells. This further substantiated our claim that the EAE selectively inhibits drug-resistant NSCLC cells more effectively than their wild-type counterparts. Solvent extracts from medicinal plants rich in various bioactive molecules, like flavonoids, alkaloids, *etc.*, have been shown to modulate EGFR-signalling pathways and subsequently cause cell cycle arrest and/or regression of cancer growth (Chun et al., 2013; Wang et al., 2018). In this context, our findings are in line with the existing literature on the potential ability of the plant extracts to exert selective inhibitory effects on the EGFR_T790M (Tang et al., 2021).

About the mechanism of inhibition, the flow cytometric analysis presented here suggested that the EAE treatment induced cell cycle arrest at the G1 phase. This cell cycle arrest indicates the EAE-mediated antiproliferative effect of EAE and is in complete agreement with the observed reduction in viability. Further, EAE treatment increased apoptosis, as evidenced quantitatively by Annexin/PI assay and qualitatively by TUNEL assay. The whole *R. graveolens* extract has been used in other cancer cells to understand its anticancer properties and it was found to induce apoptosis by interfering with p53, AKT pathways and causing the cell cycle arrest at G2/M phase (Wu et al., 2022). Studies were conducted on different lung cancer cell lines, including H1975 cells, in which ethanol extract of *Scutellaria baicalensis* and triterpenes extracted from *H. diffusa* effectively induced apoptosis by inactivating STAT3 in EGFR-TK-resistant cells (Park et al., 2021). These studies reveal that the active metabolite from plant extracts can combat different cancers, including drug-resistant lung cancer, and cause apoptosis. Our results suggested that the EAE treatment causes cell cycle arrest, inhibiting cell proliferation, and activating programmed cell death in drug-resistant NSCLC cells. This is consistent with previous studies, where alkaloid and polyphenolic compounds in medicinal plant extracts induced increased apoptosis (Ge et al., 2023; Elkady, 2013; Ratheesh, Sindhu, and Helen, 2013; Stalikas, 2007). These findings further support the multi-target therapeutic ability of natural products in cancer therapy. Next, to investigate the molecular basis for EAE-mediated inhibition of cell cycle arrest and/or activation of apoptosis, western blot analyses were performed on lysates prepared from EAE-treated cells. Here, dose-dependent suppression of phosphorylation at Tyr1068 and Tyr1173 residues of EGFR_TK was demonstrated, which was relatively higher and consistent between the experimental replicates in EAE-treated H1975 cells. These results were in order of the previous study by Wang et al. (2018), Liu and Gao (2020), Jiang et al. (2017), Qin et al. (2020), showing EGFR and pEGFR 1068 inhibition, Tang et al. (2016), Perera et al. (2005), van Rosenburgh et al. (2022) shown p1173 inhibition. The inhibition of EGFR phosphorylation at these sites agrees with a better inhibitor of EGFR_T790M.

Previous studies have shown that phosphorylated EGFR at Tyr1068 plays a crucial role in (i) recruitment of growth factor receptor-bound protein 2 (GRB2), an adapter protein (Okutani et al., 1994; Rojas et al., 1996; Yamauchi et al., 1997), which is pivotal in the activation of the Ras-MAPK signaling pathway in response to epidermal growth factor (EGF) (Sebastian et al., 2006), and (ii) serves as a docking site for proteins with the SH2 domain of signal transducer and activator of transcription 3 (STAT3) (Shao et al., 2003). Similarly EGFR phosphorylated at Tyr1173 facilitates the binding of SHC protein (Okabayashi et al., 1994). So it is well-established that the inhibition of phosphorylation at these key sites effectively disrupts EGFR signaling, thereby the viability and proliferation of NSCLC cells (Tang et al., 2021; Wang et al., 2018; Ma et al., 2020; Liu and Gao, 2020; Jiang et al., 2017; Qin et al., 2020; Tang et al., 2016; Perera et al., 2005; van Rosenburgh et al., 2022), Also, inhibition of phosphorylation of EGFR_TK was reported after treatment with plant-derived bioactive metabolites, like curcumin (Starok et al., 2015; Zhen et al., 2014), resveratrol (Wang et al., 2020), *etc.*

Further, pull-down assays using EGFR_TK and EGFR_T790M in the presence of EAE, followed by mass spectrometric analyses of the metabolic content, were performed to identify metabolite content unique to the mutant EGFR. Analysis of the data suggested the identification of a variety of bioactive metabolites belonging to different classes of phytochemicals, including alkaloids, polyketides, *etc.* Computational analyses of these metabolites for drug-like properties enabled us to refine the list of metabolites, which have now formed the basis for further investigation and validation of their therapeutic potential. Prior literature has also reported on the selective kinase inhibitory potential of these secondary metabolites, further validating our study outcomes (Gowtham et al., 2024; Marston, 2011). Sambo et al., have shown the effective inhibition of kinase activity and *in vitro* downregulation of protein kinases in lung cancer cell lines by identifying known anticancer metabolites of *Ziziphus mucronata* (Sambo et al., 2025).

*Ruta graveolens* is a well-studied plant against various ailments such as infectious diseases, neurological disorders, inflammatory diseases, cardiovascular diseases, gastrointestinal diseases, metabolic diseases, dermatological conditions, etc .,(Ratheesh et al., 2013; Jinous, 2012; Eickhorst et al., 2007). It is rich in alkaloids, flavonoids, coumarins, and essential oils, which are known to exert antimicrobial, anti-inflammatory, antioxidant, and anticancer properties (Jinous, 2012). With specific regard to cancer treatment, extracts from at least four of the above-cited Ruta species have been investigated for their potential against colon cancer, breast cancer, lung cancer, leukemia, *etc.*, (Fadlalla et al., 2011; Gentile et al., 2015; Varamini, Soltani, and Ghaderi, 2009; Richardson et al., 2016; Pathak et al., 2003; Arora and Tandon, 2015; Pushpa et al., 2015; Schelz et al., 2016; Fuselier et al., 2023). Mechanistic studies attributed their anticancer potential to the efficient induction of apoptosis, inhibition of cell proliferation, and induction of oxidative stress (Gentile et al., 2015; Freyer et al., 2014). Among Ruta species, *R. graveolens* has been extensively investigated as cancer therapeutics, including in clinical trials (Gentile et al., 2015; Freyer et al., 2014). In one such trial, Ruta was given with Ca_3_(PO_4_)_2_ to treat brain cancers, particularly gliomas. As part, respective brain cancer cells were treated with the Ruta extracts *ex vivo* and injected into human patients. This resulted in complete regression of tumors in six out of seven glioma patients (Pathak et al., 2003).

The present study’s findings strongly supported the therapeutic potential of EAE from *R. graveolens* in treating NSCLC, particularly in those carrying the EGFR_T790M mutation. The multiple inhibitory mechanisms exerted by EAE from *R. graveolens*, including cell cycle disruption, apoptosis induction, and direct inhibition of EGFR phosphorylation, highlight its promise as a natural anticancer agent. Future studies should focus on isolating and characterizing the active metabolites from the EAE preparation from the leaves of *R. graveolens* to refine and enhance their efficacy as EGFR_T790M mutant-specific NSCLC therapeutics. Additionally, *in vivo* studies and clinical evaluations are warranted to translate the findings of this study into potential treatment strategies for patients with drug-resistant NSCLC cancer. The alignment of our results with prior research on plant-based kinase inhibitors highlights the growing relevance of natural products in anticancer drug discovery.

## Data Availability

The raw data supporting the conclusions of this article will be made available by the authors, without undue reservation.

## Abbreviations

EAE, Ethyl Acetate Extract; EGFR, Epidermal Growth Factor Receptor; EGFR_TK, EGFR Tyrosine Kinase (wild-type); EGFR_T790M, Substitution of threonine (T) with a methionine (M) at position 790 of exon 20 of EGFR; SCLC, Small-Cell Lung Cancer; NSCLC, Non-Small Cell Lung Cancer; TKI, Tyrosine Kinase Inhibitors; FDA, Food and Drug Administration; DMSO, Dimethyl Sulfoxide; TUNEL, Terminal deoxynucleotidyl transferase [TdT] dUTP Nick-End Labeling.
