# Supplementary material for: Ethyl acetate extract of Ruta graveolens: a specific and potent inhibitor against the drug-resistant EGFR_T790M mutant in NSCLC
Source: Front Pharmacol. 2025 Apr 29;16:1570108. doi: 10.3389/fphar.2025.1570108 (PMC12069052; doi:10.3389/fphar.2025.1570108)
Supplement: Supplementary file 2 [file DataSheet1.docx]

**Figure S1.** EGFR_TK and EGFR_T790M Kinase assay optimization

| EGFR_TK protein activity assay | 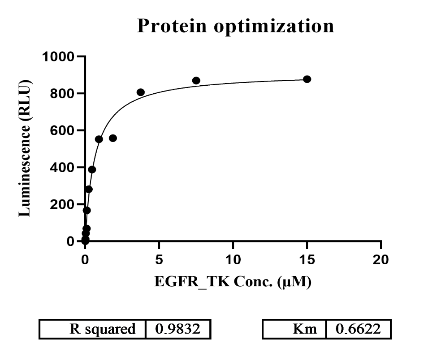 | 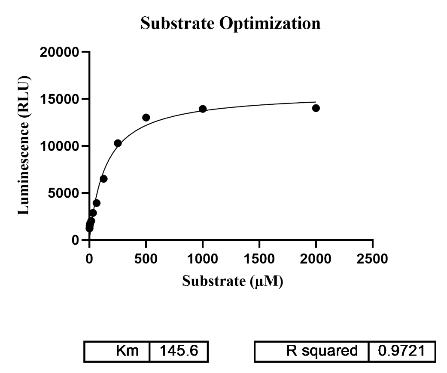 | 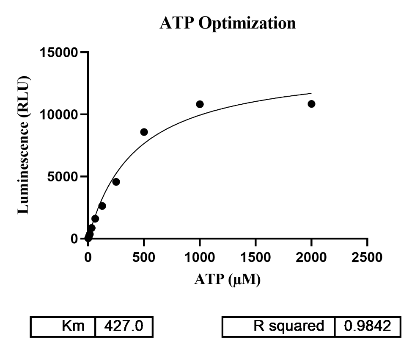 |
| --- | --- | --- | --- |
|  | **Protein optimization**  **Proein 0 - 15µM**  Substrate - 10µM  ATP - 500µM | **Substrate optimization**  **Substrate - 0-2 mM**  Protein - 4 µM  ATP - 1.5mM | **ATP optimization**  **ATP 0 – 2mM**  Protein - 4 µM  Substrate - 500µM |
| EGFR_T790M Protein Activity  assay | 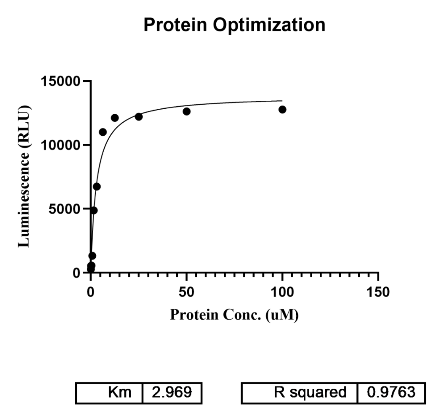 | 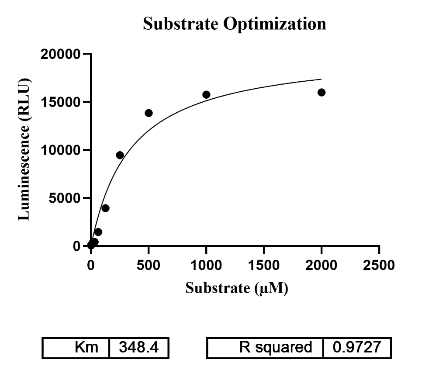 | 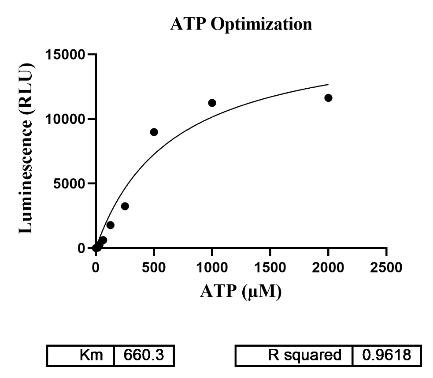 |
|  | **Protein optimization Protein – 0- 100µM**  Substrate - 600µM  ATP – 1mM | **Substrate optimization Substrate - 0-2 mM**  Protein - 6 µM  ATP - 1mM | **ATP optimization**  **ATP 0 – 2mM**  Protein - 6 µM  Substrate - 600µM |

**Figure S2a.** LC-ESI-MS/MS (Positive ion mode) Chromatogram of crude extract of *R. graveolens*

**Figure S2b.** LC-ESI-MS/MS (Negative ion mode) Chromatogram of crude extract of *R. graveolens*

**Figure S3a.** LC-ESI-MS/MS (Positive ion mode) Chromatogram of EGFR_T790M-EAE

**Figure S3b.** LC-ESI-MS/MS (Negative ion mode) Chromatogram of EGFR_T790M-EAE

**Figure S4a.** LC-ESI-MS/MS (Positive ion mode) Chromatogram of EGFR-TK-EAE

**Figure S4b.** LC-ESI-MS/MS (Negative ion mode) Chromatogram of EGFR-TK-EAE

**Table S1.** Compounds with drug-like properties specific to EGFR_T790M

| **S. No.** | **Compound Name** | **ADME properties** | **Class** | **MS/MS Match Percent** |
| --- | --- | --- | --- | --- |
|  | Balticol F | H-bond acceptors – 6  H-bond donors – 2  iLOGP - 2.5  Lipinski violations - 0 | Naphthalenone derivative | 95.71 |
|  | 4,7,8-trimethoxyfuro[2,3-b]quinoline | H-bond acceptors – 5  H-bond donors – 0  iLOGP – 2.78  Lipinski violations - 0 | Alkaloid | 94.9 |
|  | Communesin A | H-bond acceptors – 3  H-bond donors – 1  iLOGP – 3.62  Lipinski violations - 0 | Alkaloids | 93.17 |
|  | Talaroenamine D | H-bond acceptors – 5  H-bond donors – 1  iLOGP – 1.7  Lipinski violations - 0 | Benzoyl derivatives | 91.4 |
|  | Thailanstatin A | H-bond acceptors – 9  H-bond donors – 3  iLOGP – 3.02  Lipinski violations - 0 | Polyketide | 87.93 |
|  | Chryxanthone B | H-bond acceptors – 6  H-bond donors – 2  iLOGP – 3.56  Lipinski violations - 0 | Xanthone | 87.11 |
